# Activation of Coke Fines Using CO_2_ and Steam: Optimization and Characterization of Carbon Sorbents

**DOI:** 10.3390/molecules30122528

**Published:** 2025-06-10

**Authors:** Aigul T. Ordabaeva, Zainulla M. Muldakhmetov, Mazhit G. Meiramov, Sergey V. Kim

**Affiliations:** Institute of Organic Synthesis and Chemistry of Coal of Kazakhstan Republic, Alikhanov Str., 1, Karaganda 100012, Kazakhstanvanquishv8@mail.ru (S.V.K.)

**Keywords:** coke fines, activation, sorbent

## Abstract

In this study, the characteristics of coal sorbents obtained by the activation of coke fines in an atmosphere of a mixture of gases CO_2_ and H_2_O were studied. The experiment was conducted at various temperatures (700–900 °C), activation time (60–180 min), and constant CO_2_ supply rate (0.5 L/min). The main parameters such as tinder, ash content, bulk density, sorption capacity, total pore volume, and specific surface area were analyzed to assess the efficiency of the process. The results showed that samples of sorbents obtained at a temperature of 800 °C and an activation time of 120 min have the highest sorption capacity for iodine (up to 64.77%). The specific surface area of the obtained carbon sorbents was ~432.6 m^2^/g. It was found that an increase in temperature to 900 °C leads to a decrease in sorption characteristics, which may be due to partial destruction of the porous structure of the material. It was also found that the duration of activation contributes to an increase in burn-off and ash content, which had an effect on sorption properties. Based on the data obtained, optimal conditions for the production of carbon sorbents have been established and a process model has been developed.

## 1. Introduction

One of the most urgent tasks of our time is the development of efficient and environmentally friendly technologies for water purification from pollutants [1,2,3,4,5]. Special attention is paid to the removal of pollutants that have a negative impact on the environment and human health. In addition to liquid waste, the development of gas purification and gas storage systems using activated carbons is also an important area of research [6,7,8]. To solve these problems, various methods are actively being investigated, among which adsorption on carbon materials occupies one of the leading places due to its simplicity, cost-effectiveness, and high efficiency [9].

In addition to purification, activated carbon can be used as a catalyst carrier [10,11], as well as to produce supercapacitors [12,13,14].

A wide variety of materials are used as raw materials for the production of sorbents, such as biomass, fossil coals, waste from various industries, etc. [15,16,17,18,19].

Despite significant progress in this field, there are still open questions regarding the effect of the type of feedstock, activating agent, and optimal conditions for obtaining carbon sorbents on their adsorption properties, as well as on the mechanisms of interaction with various types of pollutants [20,21,22,23,24,25].

CO_2_ is an effective activating agent that is widely used to produce carbon sorbents. The activation of various types of materials in a CO_2_ atmosphere makes it possible to obtain materials with a developed porous structure and high values of specific surface area and sorption capacity.

For example, Lubchik et al. [26] showed that the activation of anthracite, previously modified with perchloric acid (HClO_4_), in a CO_2_ atmosphere at a temperature of 850 °C makes it possible to obtain activated carbons with a specific surface area of up to 1600 m^2^/g with a burning degree of about 70%. It is noted that the samples obtained by this approach are characterized by a well-balanced microporous and mesoporous structure.

Recent studies show that not only the specific surface area but also the surface functional groups and pore types play a crucial role in the sorption of organic pollutants. For example, activated carbon derived from Eucalyptus globulus seeds using ZnCl_2_, even with a moderate surface area (250–300 m^2^/g), demonstrated high efficiency in phenol adsorption due to the predominance of microporosity and the presence of basic groups on the surface [27].

Research by Kunarbekova et al. [28] demonstrates the high efficiency of carbon materials obtained from biomass and activated with KOH, with a specific surface area exceeding 2000 m^2^/g and an iodine sorption capacity of up to 6.46 g I_2_/g. Such sorbents can be used for the removal of radioactive iodine at nuclear industry sites.

Activation of carbon materials using CO_2_ and steam is also employed to produce activated carbons. However, these two gases affect the material structure differently. Studies [29] have shown that during activation, CO_2_ and H_2_O compete for the same active sites on the carbon surface, which may lead to the inhibition of one reaction by the other. The addition of CO_2_ to a steam activation system results in a decrease in the reaction rate of steam, indicating partial inhibition of the steam–carbon interaction. The study also found that CO_2_ interacts with carbon predominantly via the reaction C + CO_2_ → 2CO, which occurs on the internal pore surfaces and contributes to the development of a microporous structure.

In the work of Zhang et al. [30] it was found that the activation of coal obtained by pyrolysis of wood and agricultural waste (oak, corn husks, and stems) in a CO_2_ atmosphere at 800 °C for 1–2 h makes it possible to obtain activated coals with a specific surface area of up to 1010 m^2^/g and a predominantly microporous structure. Acevedo et al. [31] showed that activated carbons with high values of sorption capacity for Congo red dye (83–98%) and specific surface area (up to 208–991 m^2^/g) can be obtained from tire waste and their mixtures with brown coal and bituminous waste by activation in a CO_2_ atmosphere at temperatures up to 850 °C.

In addition to CO_2_, steam activation (H_2_O) is used to produce activated carbons, which allows for the production of activated carbons with a developed surface and porosity. In Zhou [32] et al., it has been shown that steam activation of tea production waste provides activated carbons with a specific surface area of up to 995 m^2^/g at a temperature of 800 °C and an activation time of 0.5 h; however, samples with a specific surface area of 563.2 m^2^/g obtained at 750 °C have the best balance between micro- and mesopores.

In our previous study [33], activated carbon was obtained by steam activation of coke fines at 850 °C for 120 min, with a specific surface area of ~301 m^2^/g and an iodine sorption capacity of 26.75%.

The combined effect of steam and CO_2_ during high-temperature processing of various materials also makes it possible to obtain activated carbons with high sorption capacity. So, in the article by Minkova et al. [34], a high sorption capacity for iodine (565 mg/g) was demonstrated by activated carbon samples obtained by activating sugar cane waste with a mixture of water vapor and CO_2_ at a temperature of 750 °C with an activation time of 2 h.

The purpose of this study is to study the effect of various parameters of activation of coke fines in a mixture of CO_2_ and H_2_O gases on the characteristics of the obtained carbon sorbents. The results of the work are aimed at identifying optimal conditions for the production of sorbents that can be used to purify water from pollutants.

The main result of our study was the establishment that sorbents with a maximum sorption capacity of iodine (up to 64.77%) are obtained at a temperature of 800 °C and an activation time of 120 min. The specific surface area obtained was ~432.6 m^2^/g. The response surface methodology (RSM) method was used to develop the process model.

## 2. Results

Experimental values of iodine capacity (*q_Iod_*, %) were obtained with different combinations of factors. Table 1 shows the experimental plan (factors and their levels) and the obtained iodine capacity values (*q_Iod_*, %).

The coefficients of the regression model are determined based on the results of mathematical processing of experimental data. Table 2 contains the values of the coefficients of the model and their statistical evaluation.

The resulting mathematical model of the process looks like this:

(1) $$q_{Iod}=65.19-2.54T+4.23\tau -15.24T^{2}-8.89\tau^{2}-8.57T\tau$$

The significance of the regression coefficients was assessed using the Student’s *t*-test, where the standard error of each coefficient was calculated based on the diagonal elements of the inverse matrix (*X^T^X*)^−1^ and the variance of the model error.

The standard error (*SE*) of each regression coefficient was calculated using the square root of the corresponding diagonal element of the inverse matrix (*X^T^X*)^−1^ multiplied by the standard error of the model(s). Then the Student’s *t*-test was applied to determine the significance of each coefficient.

In Table 2, the *p*-value is the probability of obtaining the observed data (or more extreme ones), assuming that the null hypothesis is correct.

A comparison of experimental (quad experiment, %) and calculated iodine capacity values (*q_Iod_*, %) is shown in Table 3.

To check the quality of the model, the coefficient of determination *R*^2^ was calculated, which was 0.97, which indicates a high degree of consistency between experimental and calculated response values.

Coefficient of determination *R*^2^ according to Formula (4): $R^{2}=1-\frac{SS_{res}}{SS_{tot}}=1-\frac{34.01}{1096.77}$ ≈ 0.97.

Error variance (*S*^2^), calculated by Formula (8): $S^{2}=\frac{\sum_{i=1}^{n}{{(Y}_{exp,\;i}-Y_{pred,i})}^{2}}{n-p}$ = $\frac{34.01}{9-6}=$11.34.

According to the experimental design (Table 1), the number of experimental data points was *n* = 9, and the number of model coefficients (*β_i_*) in the regression equation was *p* = 6. The root mean square error *S* was calculated using Equation (9): $S=\sqrt{S^{2}}=\sqrt{11.34}$ = 3.37.

The sum of squares due to the model, according to Equation (10), was calculated as: $SS_{mod}=SS_{tot}-SS_{res}$ = 1096.77 − 34.01 = 1062.77.

The results of the analysis of variance (ANOVA) confirming the adequacy of the constructed model are shown in Table 4.

Based on the results of the analysis of variance (Table 4), it was established that the constructed model is statistically significant, as the calculated value of the *F*-statistic (18.75) exceeds the critical value *F_crit_* at a significance level of *α* = 0.05. This confirms the adequacy of the model and its suitability for describing the studied process.

The calculated *F*-statistic (*F*), obtained using Equation (11), was: F=SSmodpSSres(n−p)=1062.77534.013 = 18.75.

The number of degrees of freedom of the numerator (*df*_1_), using Formula (13), was *df*_1_ = *n* − *p* = 9 − 6 = 3, where *n* = 9 (number of experimental points), *p* = 6 (number of coefficients in the model).

The number of degrees of freedom of the denominator (*df*_2_), according to Formula (14), was *df*_2_ = *p* − 1 = 6 − 1 = 5, where *p* = 6 (the number of coefficients in the model). Thus, we find by Formula (12): *F_crit_* = *Q_F_*(3; 5; 0.05) = 5.41 (at the standard value of the significance level: *α* = 0.05).

Thus, we obtained that *R*^2^ = 0.97, *F* (18.75) > *F_crit_* (5.41); therefore, the obtained model is adequate.

The accuracy of the model is confirmed by a small value of the standard deviation of the error (*S* = 3.37).

**Figure 1:**
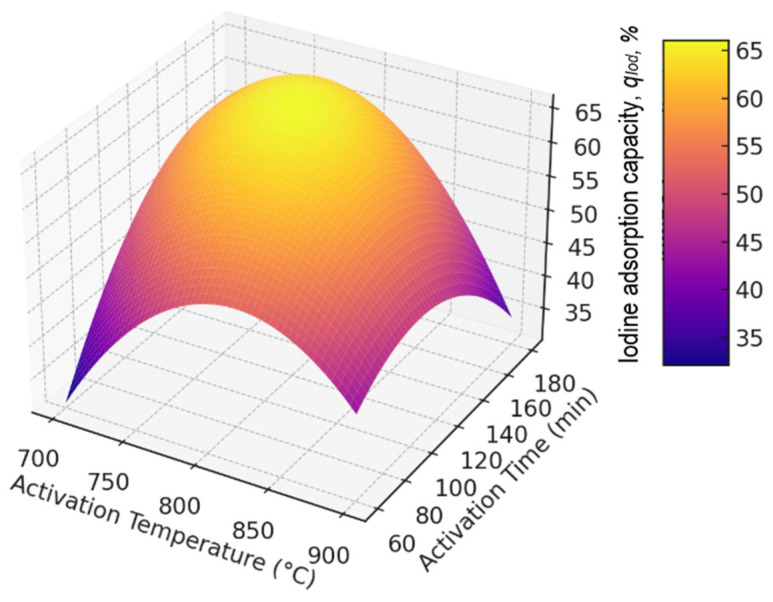
Response surface plot.

The response surface plot is shown in Figure 1.

It can be seen from the graph in Figure 1 that the maximum response is observed near 800 °C with an activation time of 120 min, which confirms the experimental values. When the temperature rises to 900 °C or the time increases to 180 min, a decrease in sorption capacity is observed. It is also seen that the response decreases sharply at low temperatures and short activation times.

**Figure 2:**
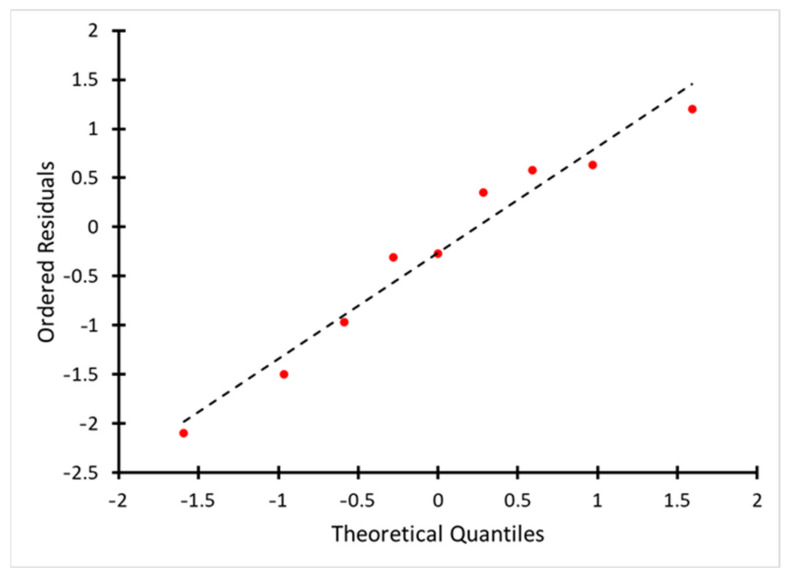
Normal plot of residuals.

The normal plot of residuals is shown in Figure 2.

**Figure 3:**
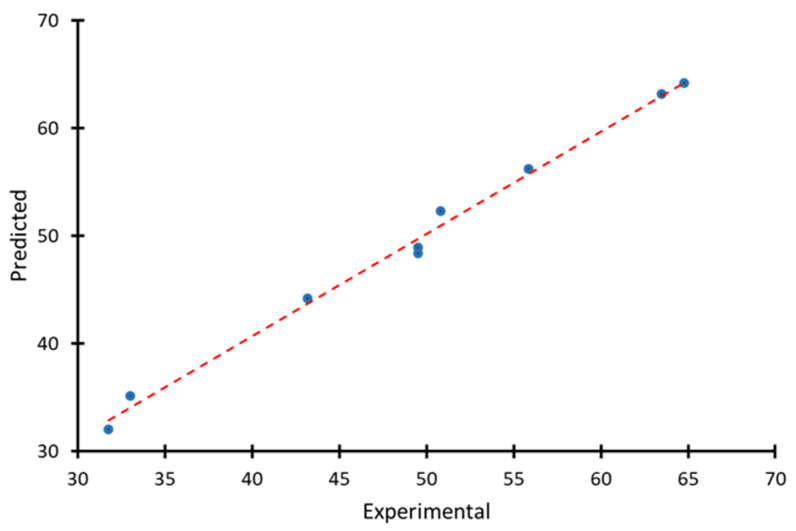
Plot of predicted vs. experimental values.

The graph of the normality of the residuals in Figure 3 confirms that the remnants of the model are distributed close to the normal law, which makes the model statistically justified and allows the correct use of significance criteria in the analysis of the response surface.

The plot of predicted vs. experimental values is shown in Figure 3.

Figure 3 shows that all the points are located very close to the diagonal, which indicates the high accuracy of the model’s predictions. It can also be seen that there are no systematic deviations, the points are evenly distributed along the line, and there is no displacement or curvature. A possible partial overlap in the range of 49–50 indicates repeated or very close values in the experiment.

The samples obtained at *T* = 800 °C and *τ* = 120 min showed the highest adsorption capacity for iodine (*q_Iod experiment_* = 64.77%). The specific surface area of these samples, measured by the BET method, was ~432.6 m^2^/g. The specific surface area of the initial material, coke fines, was ~14.7 m^2^/g.

**Figure 4:**
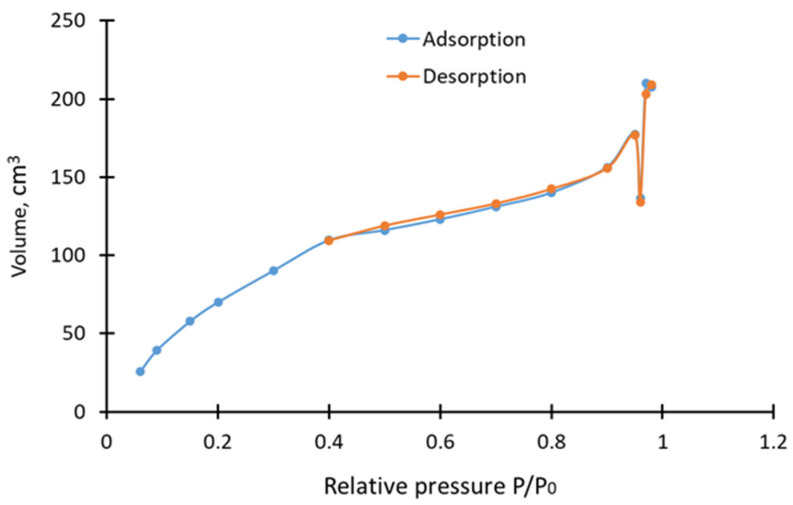
Isotherm of nitrogen adsorption by the obtained carbon sorbent.

The isotherm of nitrogen adsorption by the resulting carbon sorbent is shown in Figure 4.

As a result of the BET analysis, it was also found that in the resulting carbon sorbent, the pore volume with *R* less than 47.7 nm is 0.323 cm^3^/g. For coke fines, this indicator was 0.039 cm^3^/g. The distribution of pores relative to their total volume is shown in Table 5.

As can be seen from Table 5, the resulting sorbent has a significantly larger pore volume and specific surface area. The analysis of the pore distribution relative to the total volume shown in Table 5 showed that the main contribution to the total pore volume is made by pores with a diameter of about 56.122 nm, totaling approximately 63.18% of the total volume. Pores with diameters of about 3.4957 nm, 4.4297 nm, and 5.8631 nm (totaling about 31.67% of the total volume) also make a significant contribution. Table 5 shows that there is a small number of pores (5.15%) with a diameter of 79.642 nm.

**Figure 5:**
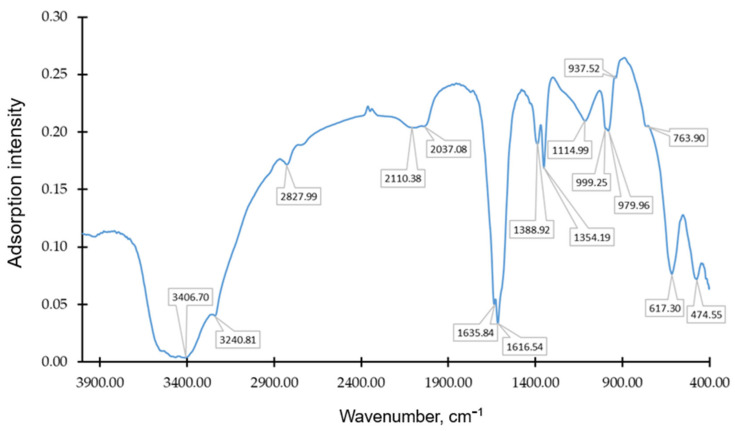
The IR spectrum of the resulting activated carbon.

The IR spectrum of the resulting carbon sorbent is shown in Figure 5.

The low-frequency peak of 474.55 cm^−1^ in Figure 2 may be associated with deformation fluctuations of various carbon structures or impurities, such as metal oxides. The region of about 600–700 cm^−1^ is often associated with deformational fluctuations of C-H in aromatic structures. This peak may also indicate the presence of mineral impurities or oxides. The peak at 763.90 cm^−1^ may indicate C-H deformation fluctuations in aromatic compounds. Such peaks are often found in compounds with aromatic rings, which may be a sign of residual hydrocarbon structures in activated carbon. The peaks at 937.52 cm^−1^ and 979.96 cm^−1^ may indicate the presence of C-O extensions in carbon compounds or structural elements containing oxygen. In activated carbon, such peaks may appear due to surface oxidation or the presence of impurities. The peak at 999.25 cm^−1^ may also be associated with C-O fluctuations, indicating the possible presence of esters or phenolic structures. In some cases, this peak indicates the presence of impurities or oxidized areas on the surface. The peak at 1114.99 cm^−1^ may indicate the presence of oxygen-containing functional groups formed on the surface of activated carbon and is characteristic of C-O strains associated with phenolic or ester groups. Peaks with values of 1354.19 cm^−1^ and 1388.92 cm^−1^ may be associated with deformational fluctuations of C-H in methyl (CH_3_) and methylene (CH_2_) groups, which may indicate the presence of organic residues or aromatic structures. The 1600–1650 cm^−1^ region is typical for C=C oscillations in aromatic rings or graphite-like structures, so the peaks at 1616.54 cm^−1^ and 1635.84 cm^−1^ in activated carbon may indicate the presence of aromatic structures or carbon fragments with double bonds.

The range from 2000 to 2200 cm^−1^ in Figure 2 may be related to fluctuations in C≡C (alkynes) or C≡N (nitriles) bonds. In this regard, the presence of peaks at 2037.08 cm^−1^ and 2110.38 cm^−1^ may indicate the presence of small amounts of organic compounds formed as a result of heat treatment.

The region of about 2800–3000 cm^−1^ is usually associated with the stretching of C-H bonds in methyl and methylene groups (CH_3_ and CH_2_). Based on this, the peak at 2927.99 cm^−1^ in Figure 2 may indicate the presence of organic compound residues. The peaks at 3240.81 cm^−1^ and 3406.70 cm^−1^ may indicate O-H stretching, especially in the presence of hydrogen bonds. In activated carbon, this peak may indicate the presence of hydroxyl groups or adsorbed water.

According to the results of the experiments, it was found that the carbon sorbent obtained with samples of sorbents obtained at a temperature of 800 °C and an activation time of 120 min has the highest sorption capacity for iodine, *q_Iod experiment_* = 64.77%. At the same time, the ash content of the resulting carbon concentrate was 10.83%, and the bulk density was 476 g/dm^3^.

## 3. Discussion

As a result of the research, it was found that the activation of coke fines in a mixture of carbon dioxide and water vapor makes it possible to obtain a carbon sorbent with a specific surface area of ~432.6 m^2^/g and an iodine sorption capacity of 64.77%. The obtained indicators are significantly higher than those obtained from our previous study [33], when only water vapor was used to activate coke fines, and the resulting carbon sorbent had a specific surface area of ~301 m^2^/g and an iodine sorption capacity of 26.75%.

It is known that CO_2_ mainly contributes to the development of micropores, while water vapor plays a key role in the formation of meso- and macropores. In this work, the use of a mixture of gases made it possible to achieve a synergistic effect [35]: CO_2_ provided the development of a specific surface area and, possibly, microporosity, and steam contributes to the expansion of existing pores and the formation of a mesoporous structure. This is confirmed by the data of the BET analysis, according to which the bulk of the volume falls in the range of about 56 nm (~63%), but a significant contribution is also made by the mesoporous range (3–6 nm, ~31.67%)

Table 1 shows that the sorption capacity for iodine decreases at 900 °C and when the duration of the process is 120 and 180 min, and the samples obtained under these conditions have the highest charring and ash values. This may indicate a partial destruction of the porous structure of the material due to the intense burning of carbon, which can lead to the destruction of pores and the formation of nonflammable products. A high ash content may indicate that more nonflammable impurities remain in the activated material, which can clog the pores, thereby reducing the adsorption properties [36,37].

The present study did not study the effect of additional acid or alkaline treatment of the feedstock, as well as changes in the water vapor:CO_2_ ratios on the formation of micro- and mesoporous structures, sorption capacity to various kinds of pollutants, electrophysical characteristics, etc. These areas of research will be conducted in the future.

Maya et al. [38] found that by varying the ratio of CO_2_:water vapor in a gas mixture, it is possible to control the pore structure of activated carbon: an increase in the proportion of steam promotes the formation of mesopores, while the predominant CO_2_ content enhances the development of microporosity. It is also shown that with increasing temperature and degree of transformation, the porous structure collapses, the mechanism of which is associated with the intersection and fusion of pores. In addition, the presence of mineral residues can contribute to the clogging of pores and reduction in sorption properties.

The use of additional acid or alkaline treatment of the feedstock may improve the quality characteristics of the obtained sorbents. For example, Kan et al. [39] found that the impregnation of the feedstock with an H_3_PO_4_ solution before activation makes it possible to increase the proportion of micropores in the resulting activated carbon.

Acid or alkaline processing is also a promising direction, not only for the feedstock but also for the resulting sorbent. Thus, Guedidi et al. [40] found that the treatment of commercial activated carbon with an alkaline solution followed by oxidation with a solution of H_2_O_2_ under the influence of ultrasound increases the concentration of oxygen-containing groups on its surface, which improves the sorption characteristics of coal without significant loss of textural properties.

From the intensity of the peaks and the width of the IR spectroscopy bands, Figure 5 shows that the resulting sorbent is dominated by hydroxyl (-OH) functional groups, which are responsible for the high acidity and reactivity of the surface. They form hydrogen bonds and participate in the adsorption of polar pollutants [41], such as phenols, dyes, and heavy metals [2,42,43,44,45].

An analysis of the data on bulk density (376–595 g/dm^3^) and the degree of burnout (~83% at 900 °C) allows us to conclude that there is a significant loss of mass of the feedstock during the activation process. This may be due to the intense interaction of carbon with activating agents (CO_2_ and H_2_O), which leads to the formation of pores and the removal of volatile components.

At the same time, the maximum adsorption capacity for iodine (64.77%) is achieved with a moderate degree of burnout (~46%), corresponding to a temperature of 800 °C and an activation time of 120 min. Thus, it is these conditions that provide the optimal balance between weight loss and the development of a porous structure.

The results of the study showed that a further increase in temperature to 900 °C is impractical since there is a decrease in adsorption activity and an increase in ash content, which negatively affects the quality of the final product.

**Figure 6:**
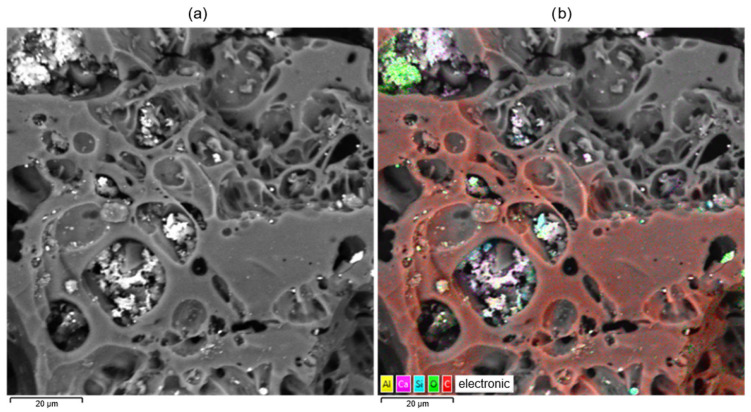
SEM image and EDS image: (**a**)—SEM; (**b**)—EDS.

The analysis of electron microscopy (SEM) and energy dispersion spectroscopy (EDS) is shown in Figure 6.

**Figure 7:**
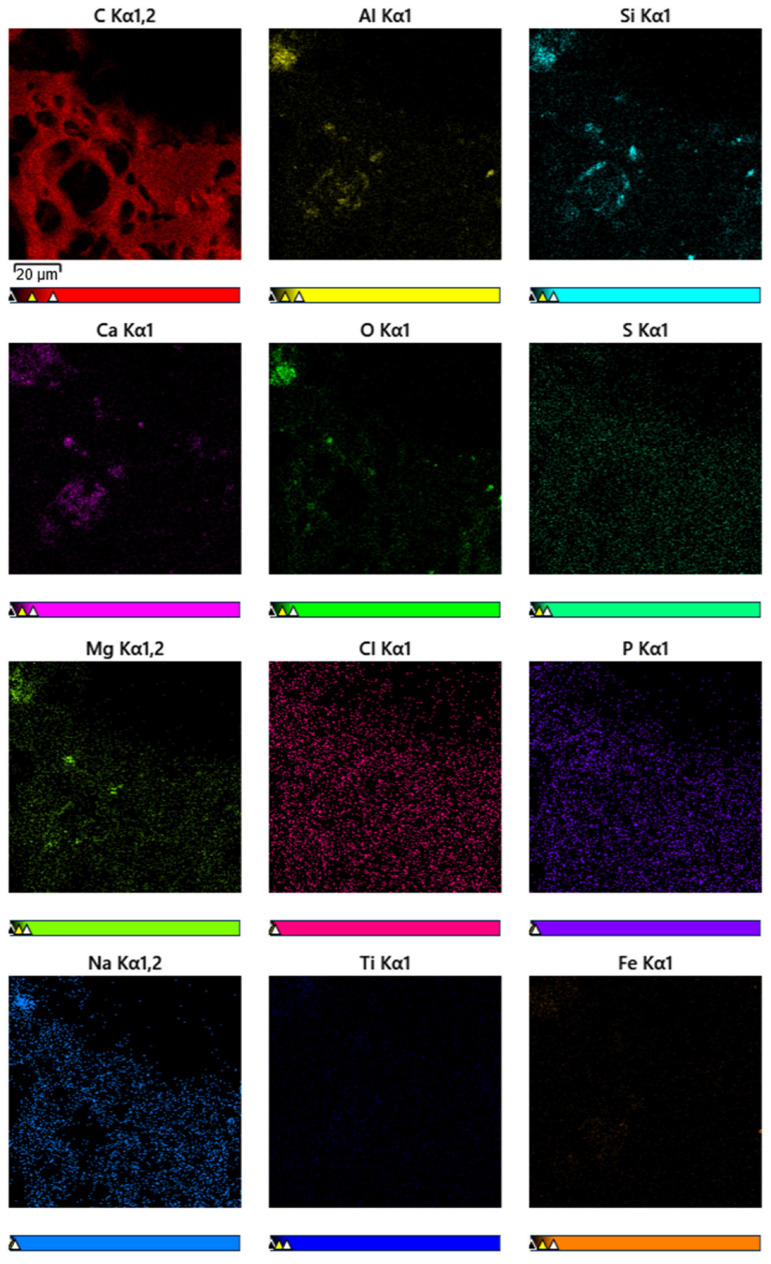
Element mapping.

The element mapping is shown in Figure 7.

Figure 6 shows the microstructure of the sorbent obtained at a temperature of 900 °C. As can be seen from Figure 6, light inclusions are present in the pores of the material, morphologically different from the carbon matrix. Based on the energy dispersion analysis (EDS) shown in the accompanying image, it can be concluded that these inclusions consist mainly of the elements aluminum (Al), silicon (Si), calcium (Ca), and sulfur (S). This elemental composition is typical for the residual mineral phase formed during the activation or heat treatment of the feedstock. These components appear to be ash formations localized in large pores of the carbon material.

## 4. Materials and Methods

To obtain the sorbent, coke fines of class 0–10 mm were used, which were crushed on a hammer crusher followed by fractionation on a vibrating stand to obtain fractions of 2–5 mm in size. Next, the resulting fraction of coke fines was dried at a temperature of 120 °C for 3 h.

**Figure 8:**
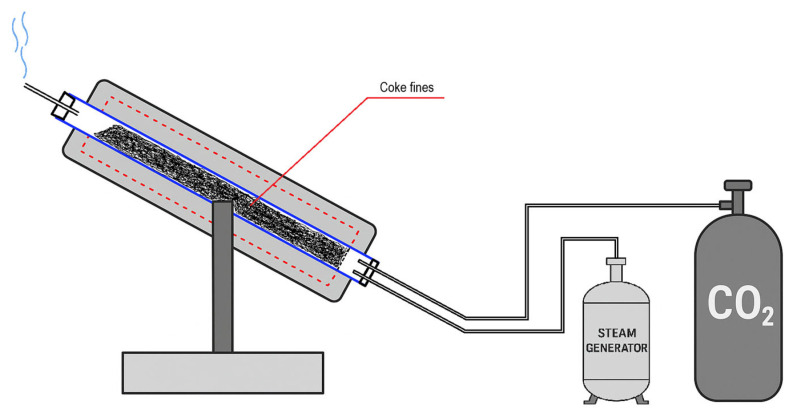
Coke fines activation setup.

The activation of coke fines was carried out on an installation of an original design that allows the activation of coke fines in the temperature range of 700–900 °C when a mixture of water vapor and carbon dioxide (CO_2_) is applied. The activation setup scheme is presented in Figure 8. The reactor core is a tube of quartz glass placed in a tube furnace RT-60-300/1200 (Henan Dming Technology Co., Ltd., Zhengzhou, China). At one end of the tubular reactor, the lid is equipped with two nozzles: one for supplying carbon dioxide, and the other for supplying water vapor. The cover of the other end of the tubular reactor is equipped with a nozzle for the discharge of gases generated during activation. Carbon dioxide was supplied to the reactor at a constant value of 0.5 L/min. The CO_2_ gas flow rate was controlled using a flow meter, and the steam supply was regulated using a steam generator heating power regulator. The amount of water used for activation was determined by the difference in water volume at the beginning and end of the experiment. The heating rate of the furnace was 20 °C/min.

After reaching the set temperature regime, the activation process was carried out for a duration in the range of 90–180 min.

To determine the optimal conditions for the activation of coke fines, an experimental plan was drawn up. The activation time (τ, min) and temperature (*T*, °C) were accepted as the main factors influencing the activation process of coke fines. The iodine adsorption capacity (*q_Iod_*, %) was used as the system response (an indicator of activation efficiency).

In this study, the response surface methodology (RSM) was used to develop a mathematical model describing the effect of activation temperature and activation time on the ability of the resulting activated carbon to adsorb iodine (*q_Iod_*, %). The RSM method is widely used for modeling and optimizing various processes [46,47,48]. It allows us to determine the optimal process conditions using a limited number of experimental cycles, which is especially important in chemical and adsorption processes due to the duration and high cost of experimental procedures. In addition, RSM makes it possible to evaluate both linear and nonlinear effects of factors and their interactions, which makes it a suitable tool for modeling complex systems where the response behavior is not strictly linear.

After conducting the experiments, according to the plan, the initial matrix [X] was compiled using the data obtained, which in general looks like this:

$$\left(X\right)=\left(\begin{array}{cccccc} 1 & x_{1,1} & x_{2,1} & x_{1,\;1}^{2} & x_{2,1}^{2} & x_{1,1}x_{2,1}\\ 1 & x_{1,2} & x_{2,2} & x_{1,2}^{2} & x_{2,2}^{2} & x_{1,2}x_{2,2}\\ \vdots & \vdots & \vdots & \vdots & \vdots & \vdots \\ 1 & x_{1,n} & x_{2,n} & x_{1,n}^{2} & x_{2,n}^{2} & x_{1,n}x_{2,n} \end{array}\right)$$

Next, the original matrix was mathematically transformed and the coefficients β were calculated using the transformed matrices using the formula:

(2) $$\hat{\beta }={\left(X^{T}X\right)}^{-1}X^{T}Y$$

where:

*X^T^*—transposed original matrix [*X*];

*X^T^X*—covariance matrix of the factors;

(*X^T^X*)^−1^—inverse matrix of the covariance matrix;

*X^T^Y*—product of the transposed factor matrix and the response vector.

As a result of the mathematical processing, the model is obtained in the form of the following equation:

(3) $$\hat{Y}=\beta_{0}+\beta_{1}x_{1}+\beta_{2}x_{2}+\beta_{3}x_{1}^{2}+\beta_{4}x_{2}^{2}+\beta_{5}x_{1}x_{2}$$

$\hat{Y}$—response;

*x*_1_—factor 1;

*x*_2_—factor 2;

*β*_0_, *β*_1_, …, *β*_5_—model coefficients.

Formula (3) allows us to find the coefficients of the regression model, minimizing the sum of the squares of the deviations between the experimental data and those calculated by the model.

### Checking the Model

The coefficient of determination (*R*^2^) is a statistical value that shows how well the regression model explains the variation of the dependent variable.

The determination coefficient is calculated according to the experimental values obtained (response values) and the values calculated according to the obtained model (Equation (3)). The formula for determining the coefficient of determination is as follows:

(4) $$R^{2}=1-\frac{SS_{res}}{SS_{tot}},$$

*SS_res_*—residual sum of squares (errors), or the sum of squared deviations (differences) between the experimental values and the values predicted by the model (Equation (2)); this value is calculated using the following formula:

(5) $$SS_{res}=\sum {\left(Y_{exp}-\hat{Y}\right)}^{2},$$

where:

*Y_exp_*—experimental values obtained from the experiment (response values);

$\hat{Y}$—values predicted by the model (Equation (3)).

*SS_tot_*—total sum of squares, representing the sum of squared deviations of the experimental data from their mean value, and is calculated using the following formula:

(6) $$SS_{tot}=\sum {\left(Y_{exp}-\bar{Y}\right)}^{2},$$

where: $\bar{Y}$—The mean value of the experimental values is calculated using the following formula:

(7) $$\bar{Y}=\frac{\sum Y_{exp}}{n},$$

where:

*n*—number of experimental values.

*R*^2^ = 1—the model fully explains the data variation (perfect prediction).

*R*^2^ = 0—the model does not explain the data variation better than the mean.

*R*^2^ < 0—the model performs worse than a simple average prediction.

The closer *R*^2^ is to 1, the better the model explains the relationship between variables.

However, a high *R*^2^ value does not always indicate model quality; it is also important to assess its adequacy and robustness.

The error variance *S*^2^ is calculated using the following formula:

(8) $$S^{2}=\frac{\sum_{i=1}^{n}{{(Y}_{exp,\;i}-Y_{pred,i})}^{2}}{n-p},$$

where:

*Y_exp,i_*—experimental values,

*Y_pred,i_*—predicted values obtained from the model,

*n*—number of experimental data points,

*p*—number of model coefficients.

According to the experimental design (Table 1), the number of experimental data points is *n* = 9, and the number of model coefficients (*β_i_*) in the regression equation is *p* = 8.

The root mean square error *S* is calculated using the following formula:

(9) $$S=\sqrt{S^{2}}$$

The sum of squares of the values predicted by the model:

(10) $$SS_{mod}=SS_{tot}-SS_{res}$$

The *F*-statistic (also known as Fisher’s *F*-statistic or *F*-test) is used in statistical analysis to evaluate the significance of a regression model. It assesses whether the model explains the variation in the dependent variable better than random chance.

The *F*-statistic is calculated using the following formula:

(11) F=SSmodpSSres(n−p),

where: *p*—number of coefficients in the regression model (Equation (3));

*n*—number of experimental data points.

The number of experimental points *n* and the number of model coefficients (*β_i_*) in Equation (3) are determined according to the experimental design.

Next, it is necessary to calculate the critical *F*-value (*F_crit_*), which is the threshold value of the *F*-statistic used to assess the adequacy of the model. The model is considered adequate if the calculated *F*-value (*F*) exceeds the critical value (*F_crit_*).

To determine the critical value *F*crit, one should refer to the Fisher distribution tables or use appropriate statistical software functions.

The general formula for calculating *F_crit_* is:

*F_crit_* = *Q_F_*(*α, df*_1_*, df*_2_**), (12)

where:

*Q_F_—*Fisher distribution function;

*α*—significance level;

*df*_1_—degrees of freedom for the numerator;

*df*_2_—degrees of freedom for the denominator.

The symbol *F_crit_* denotes the critical value of the Fisher distribution (or *F*-distribution). This statistical distribution is used to test hypotheses about the equality of variances or to assess the significance of regression models. *F_crit_* is the threshold value of the *F*-statistic used to evaluate the adequacy of the model. If the calculated value *F* > *F_crit_*, the model is considered adequate.

To calculate *F_crit_*, the following parameters are required: *df*_1_—degrees of freedom for the numerator; *df*_2_—degrees of freedom for the denominator; and the significance level *α*.

The degrees of freedom for the numerator *df*_1_ are calculated using the following formula:

*df*_1_ = *n* − *p*, (13)

where *n* is the number of experimental data points, and *p* is the number of coefficients in the model.

To determine the degrees of freedom for the denominator (*df*_2_), the following formula is used:

*df*_2_ = *p* − 1, (14)

The significance level (*α*) defines the probability of a Type I error. A commonly used standard value is *α* = 0.05.

As a result, with known values of *df*_1_, *df*_2_, and *α* = 0.05, the critical *F*-value *F_crit_* can be determined using Equation (12).

Thus, based on the analysis of the obtained values *R*^2^, *F*, and *F_crit_*, a conclusion regarding model adequacy is drawn.

The values are compared as follows:

If *F* > *F_crit_*, the model is considered adequate (the null hypothesis *H*_0_ is rejected), and the model is statistically significant.

If *F* ≤ *F_crit_*, the model is inadequate, as the predictors do not improve prediction compared to a random assumption. This indicates that the regression model does not explain the variation in the dependent variable better than a simple average or random guess.

Gas analysis was performed using gas–liquid chromatography on a Crystallux 4000 M chromatograph (RPC “Meta-chrom”, Yoshkar-OlaMeta, Russia) equipped with a dual detector module (2DTP/PID). A NaX column (3 m, d = 3 mm) was used for permanent gases, and a PoraPak R column (3 m, d = 3 mm) was used for hydrocarbon gases. The relative root mean square deviation of the detector signal (concentration) for the Crystallux 4000 M chromatograph did not exceed 2%.

Iodine activity was determined by iodometric titration. A 1 g sample of pre-dried sorbent (dried at 120 °C for 3 h) was placed in a 250 mL flask. A 0.1 mol/dm^3^ solution of iodine in potassium iodide was added, and the mixture was stirred using a magnetic stirrer for 30 min. After settling, a 10 mL aliquot of the solution was taken and titrated with a 0.1 mol/dm^3^ sodium thiosulfate solution until a pale yellow color appeared. Then, a 0.5% starch solution was added, turning the solution dark blue. Titration continued with sodium thiosulfate until the blue color disappeared.

The amount of iodine adsorbed by the sorbent was calculated using the following formula:

(15) $$X=\frac{(V_{1}-V_{2})\cdot 0.0127\cdot 100\cdot 100}{10\cdot m}$$

where:

$V_{1}$—volume of sodium thiosulfate solution used to titrate 10 mL of the iodine solution in potassium iodide;

$V_{2}$—volume of sodium thiosulfate solution used to titrate 10 mL of the iodine solution in potassium iodide after treatment with the sorbent;

0.0127—mass of iodine (in grams) corresponding to 1 mL of 0.1 mol/dm^3^ sodium thiosulfate solution;

100—volume (in mL) of the iodine-potassium iodide solution used for decolorization by the sorbent;

*m*—mass of the carbon sorbent sample, in grams.

To determine the specific surface area of the obtained sorbent samples, a low-temperature nitrogen adsorption analysis was carried out using the BET method on a Sorbi MS analyzer (Novosibirsk, Russia).

FTIR spectroscopy of the sorbents was performed using an FSM-1201 spectrometer (Infraspek, St. Petersburg, Russia) with Fspec software (version 4.0.0.2) in the transmission mode, over the wavenumber range of 400–4000 cm^−1^, with a resolution of 8.0 cm^−1^.

The ash content of the produced carbon sorbent was determined in accordance with GOST R 55661—2013 (ISO 1171:2010 Solid mineral fuels—Determination of ash (MOD)) [49].

The bulk density was determined according to the interstate standard GOST 32558–2013 (ISO 23499:2008 Coal—Determination of bulk density (MOD)) [50].

To determine the sorption capacity of a methylene blue suspension dried at 110 ° C for 2 h, the resulting sorbent weighing 0.1 g was placed in a measuring flask with a capacity of 50 cm^3^, 25 cm^3^ of a methylene blue c solution was added and shaken for 20 min. Next, the optical density of the solution was measured on a spectrophotometer (TAGLER, Moscow, Russia) at a wavelength of λ = 664 nm. The adsorption capacity of the obtained sorbent for methylene blue was determined by the formula:

(16) $$A=\frac{{(C}_{1}-C_{2})٠V}{m}$$

where *C*_1_ is the concentration of the initial methylene blue solution, mg/L; *C*_2_ is the residual concentration of the methylene blue solution after contact with the resulting sorbent, mg/L; m is the mass of the sorbent sample, g; and *V* = 0.025 is the volume of the methylene blue solution. As a result of the analysis, it was found that the sorption capacity of the resulting sorbent for methylene blue is ~120 mg/g.

## 5. Conclusions

As a result of the study, it was found that the activation of coke fines in a mixture of CO_2_ and water vapor makes it possible to obtain effective carbon sorbents with high adsorption characteristics.

Optimal conditions for sorbent production have been determined: the activation temperature is 800 °C and the process duration is 120 min. With these parameters, samples with a maximum sorption capacity for iodine (64.77%) and a specific surface area of ~432.6 m^2^/g were obtained.

Statistical analysis of the model developed using the RSM method showed its high degree of consistency between experimental and calculated values (*R*^2^ = 0.97), and statistical significance (*F* > *F_crit_*), which confirms its adequacy and suitability for describing the process under study.

It was found that an increase in temperature to 900 °C leads to a decrease in sorption characteristics due to partial destruction of the porous structure of the material and an increase in ash content.

Analysis of the porous structure showed that the main contribution to the total pore volume is made by pores with a diameter of about 56 nm (63.18% of the total volume) and pores with a size of 3–6 nm (31.67%).

The results obtained significantly exceed the indicators of the authors’ previous studies, where only water vapor was used for activation (specific surface area ~301 m^2^/g, iodine sorption capacity 26.75%).

IR spectroscopy showed the presence of various functional groups on the sorbent surface, among which hydroxyl groups predominate, which can participate in the adsorption of polar pollutants.

Thus, coke fines are a promising material for the production of highly efficient carbon sorbents.

## Figures and Tables

**Table 1:** Experimental plan and obtained experimental values of iodine capacity (*q_Iod experim__ent_*, %).

Factor 1	Factor 2	Response	Burn-Off, %	Ash, %	Bulk Density, g/dm^3^
Temperature, *T* °C	Activation Time, *τ*, min	Iodine Capacity *q_Iod_ _experiment_*, %			
700	180	55.88	29.51	9.36	537
700	120	50.8	23.93	6.41	538
700	60	33.02	17.56	7.02	595
800	180	63.5	64.88	15.17	376
800	120	64.77	46.36	10.83	476
800	60	49.53	28.97	7.20	539
900	180	31.75	83.09	26.27	411
900	120	49.53	77.62	22.58	427
900	60	43.18	46.42	9.71	510

**Table 2:** Coefficients of the obtained model and their statistical evaluation.

**Coefficient**	**Estimate (*βᵢ*)**	**Standard Error SE (*βᵢ*)**	***t*-Value**	***p*-Value**
*β*_0_	65.19	2.52	25.85	0.0001
*β*_1_	−2.54	1.39	−1.83	0.165
*β*_2_	4.23	1.39	3.04	0.056
*β*_3_	−15.24	2.38	−6.4	0.0077
*β*_4_	−8.89	2.38	−3.73	0.0336
*β*_5_	−8.57	1.69	−5.08	0.0133

**Table 3:** Comparison of Experimental and Predicted Iodine Adsorption Capacity Values (*q_Iod experiment_*, %) and Residual Errors.

Temperature (*T*), °C	Activation Time (*τ*), min	Experimental *q_Iod experiment_*, %	Predicted *q_Iod_*, %	Residual (Error), %
700	180	55.88	56.19	−0.31
700	120	50.8	52.3	−1.5
700	60	33.02	35.12	−2.1
800	180	63.5	63.15	0.35
800	120	64.77	64.19	0.58
800	60	49.53	48.9	0.63
900	180	31.75	32.02	−0.27
900	120	49.53	48.33	1.2
900	60	43.18	44.15	−0.97

**Table 4:** Results of the analysis of variance (ANOVA) for the obtained model.

Source of Variation	Sum of Squares (*SS*)	Degrees of Freedom (*df*)	Mean Square (*MS*)	*F*-Statistic	*p*-Value
Model	1062.76	5	212.55	18.75	0.018
Residual (Error)	34.01	3	11.34	—	—
Total	1096.77	8	—	—	—

**Table 5:** Distribution of pores relative to their total volume.

Coke Fines				Obtained Sorbent			
Di, nm	dDi, nm	dVi, cm^3^	dVi/Vsum, %	Di, nm	dDi, nm	dVi, cm^3^	dVi/Vsum, %
3.496	0.76746	0.00097	4.4951	3.496	0.767	0.027	14.65
4.43	1.1007	0.0019	8.7793	4.43	1.101	0.018	9.892
5.863	1.7661	0.00095	4.3632	5.863	1.766	0.013	7.128
8.441	3.389	0.00169	7.788	8.441	3.389	0	0
14.998	9.725	0.00047	2.1774	14.998	9.725	0	0
29.351	18.982	0	0	29.351	18.982	0	0
43.558	9.4333	0	0	43.558	9.433	0	0
56.122	15.694	0.01392	64.23	56.122	15.694	0.116	63.18
79.642	31.345	0.00177	8.1675	79.642	31.345	0.009	5.15

## Data Availability

Data are contained within the article.

## References

[1] El-Naas M.H., Al-Zuhair S., Abu Alhaija M. (2010). Removal of phenol from petroleum refinery wastewater through adsorption on date-pit activated carbon. Chem. Eng. J..

[2] Cho D.-W., Chon C.-M., Kim Y., Jeon B.-H., Schwartz F.W., Lee E.-S., Song H. (2011). Adsorption of nitrate and Cr(VI) by cationic polymer-modified granular activated carbon. Chem. Eng. J..

[3] Mansour F., Al-Hindi M., Yahfoufi R., Ayoub G.M., Ahmad M.N. (2017). The use of activated carbon for the removal of pharmaceuticals from aqueous solutions: A review. Rev. Environ. Sci. Biotechnol..

[4] Fröhlich A.C., dos Reis G.S., Pavan F.A., Lima É.C., Foletto E.L., Dotto G.L. (2018). Improvement of activated carbon characteristics by sonication and its application for pharmaceutical contaminant adsorption. Environ. Sci. Pollut. Res..

[5] Benammar H.S., Guergazi S., Youcef S., Youcef L. (2021). Removal of Congo red and Naphthol blue black dyes from aqueous solution by adsorption on activated carbon: Characterization, kinetic and equilibrium in nonlinear models studies. Desalination Water Treat..

[6] Toprak A., Kopac T. (2011). Surface and hydrogen sorption characteristics of various activated carbons developed from Rat coal mine (Zonguldak) and anthracite. Chin. J. Chem. Eng..

[7] Awadallah-F A., Al-Muhtaseb S.A. (2013). Carbon dioxide sequestration and methane removal from exhaust gases using resorcinol–formaldehyde activated carbon xerogel. Adsorption.

[8] Giraldo L., Vargas D.P., Moreno-Piraján J.C. (2020). Study of CO_2_ adsorption on chemically modified activated carbon with nitric acid and ammonium aqueous. Front. Chem..

[9] Moreno-Castilla C., Rivera-Utrilla J., Carrasco-Marín F., López-Ramón M.V. (1997). On the carbon dioxide and benzene adsorption on activated carbons to study their micropore structure. Langmuir.

[10] Iwanow M., Gärtner T., Sieber V., König B. (2020). Activated carbon as catalyst support: Precursors, preparation, modification and characterization. Beilstein J. Org. Chem..

[11] Rusanen A., Kupila R., Lappalainen K., Kärkkäinen J., Hu T., Lassi U. (2020). Conversion of xylose to furfural over lignin-based activated carbon-supported iron catalysts. Catalysts.

[12] Ciftyurek E., Bragg D., Oginni O., Levelle R., Singh K., Sivanandan L., Sabolsky E.M. (2018). Performance of activated carbons synthesized from fruit dehydration biowastes for supercapacitor applications. Environ. Prog. Sustain. Energy.

[13] Lee U.-W., Yang G., Park S.-J. (2021). Improvement of mesoporosity on supercapacitive performance of activated carbons derived from coffee grounds. Bull. Korean Chem. Soc..

[14] Zou Y., Wang H., Zhang Y., Liu Y., Zhao Y., Liu Z. (2023). Synergistic effect of CO_2_ and H_2_O co-activation of Zhundong coal at a low burn-off rate on high performance supercapacitor. Fuel.

[15] Lillo-Ródenas M.A., Lozano-Castelló D., Cazorla-Amorós D., Linares-Solano A. (2001). Preparation of activated carbons from Spanish anthracite. II. Activation by NaOH. Carbon.

[16] Tsyntsarski B., Marinov S., Budinova T., Yardim M.F., Petrov N. (2013). Synthesis and characterization of activated carbon from natural asphaltites. Fuel Process. Technol..

[17] Hashemi Shahraki Z., Sharififard H., Lashanizadegan A. (2018). Grape stalks biomass as raw material for activated carbon production: Synthesis, characterization and adsorption ability. Mater. Res. Express.

[18] Blachnio M., Derylo-Marczewska A., Charmas B., Zienkiewicz-Strzalka M., Bogatyrov V., Galaburda M. (2020). Activated carbon from agricultural wastes for adsorption of organic pollutants. Molecules.

[19] Frikha K., Limousy L., Pons Claret J., Vaulot C., Florencio Pérez K., Corzo Garcia B., Bennici S. (2022). Potential valorization of waste tires as activated carbon-based adsorbent for organic contaminants removal. Materials.

[20] Sousa J.C., Mahamud M., Parra J.B., Pis J.J., Pajares J.A. (1993). Effect of operation variables in the obtention of tailored active carbons from coals. Fuel Process. Technol..

[21] Madadi Yeganeh M., Kaghazchi T., Soleimani M. (2006). Effect of raw materials on properties of activated carbons. Chem. Eng. Technol..

[22] Arenas E., Chejne F. (2004). The effect of the activating agent and temperature on the porosity development of physically activated coal chars. Carbon.

[23] El Qada E.N., Allen S.J., Walker G.M. (2008). Influence of preparation conditions on the characteristics of activated carbons produced in laboratory and pilot scale systems. Chem. Eng. J..

[24] Ania C.O., Parra J.B., Pis J.J. (2002). Effect of texture and surface chemistry on adsorptive capacities of activated carbons for phenolic compounds removal. Fuel Process. Technol..

[25] Gun’ko V.M., Turov V.V., Kozynchenko O.P., Nikolaev V.G., Tennison S.R., Meikle S.T., Snezhkova E.A., Sidorenko A.S., Ehrburger-Dolle F., Morfin I. (2011). Activation and structural and adsorption features of activated carbons with highly developed micro-, meso- and macroporosity. Adsorption.

[26] Lyubchik S.B., Benoit R., Béguin F. (2002). Influence of chemical modification of anthracite on the porosity of the resulting activated carbons. Carbon.

[27] De Smedt J., Soroush S., Heynderickx P.M., Arauzo P.J., Ronsse F. (2025). The feasibility of activated carbon derived from waste seaweed via molten salt activation in a eutectic mixture of ZnCl_2_-NaCl-KCl for adsorption of anionic dyes. Biomass Conv. Bioref..

[28] Kunarbekova M., Busquets R., Sailaukhanuly Y., Mikhalovsky S.V., Toshtay K., Kudaibergenov K., Azat S. (2024). Carbon adsorbents for the uptake of radioactive iodine from contaminated water effluents: A systematic review. Chem. Eng. J..

[29] Roberts D.G., Harris D.J. (2007). Char gasification in mixtures of CO_2_ and H_2_O: Competition and inhibition. Fuel.

[30] Zhang T., Walawender W.P., Fan L.T., Fan M., Daugaard D., Brown R.C. (2004). Preparation of activated carbon from forest and agricultural residues through CO_2_ activation. Chem. Eng. J..

[31] Acevedo B., Barriocanal C., Lupul I., Gryglewicz G. (2015). Properties and performance of mesoporous activated carbons from scrap tyres, bituminous wastes and coal. Fuel.

[32] Zhou J., Luo A., Zhao Y. (2018). Preparation and characterisation of activated carbon from waste tea by physical activation using steam. J. Air Waste Manag. Assoc..

[33] Ordabaeva A.T., Muldakhmetov Z.M., Gazaliev A.M., Kim S.V., Shaikenova Z.S., Meiramov M.G. (2023). Production of activated carbon from sifted coke and determination of its physicochemical characteristics. Molecules.

[34] Minkova V., Marinov S.P., Zanzi R., Bjornbom E., Budinova T., Stefanova M., Lakov L. (2000). Thermochemical treatment of biomass in a flow of steam or in a mixture of steam and carbon dioxide. Fuel Process. Technol..

[35] Sevilla M., Mokaya R. (2014). Energy storage applications of activated carbons: Supercapacitors and hydrogen storage. Energy Environ. Sci..

[36] Berger J., Siemieniewska T., Tomków K. (1976). Development of porosity in brown-coal chars on activation with carbon dioxide. Fuel.

[37] Lan X., Jiang X., Song Y., Jing X., Xing X. (2019). The effect of activation temperature on structure and properties of blue coke-based activated carbon by CO_2_ activation. Green Process. Synth..

[38] Maya J.C., Macías R., Gómez C.A., Chejne F. (2020). On the evolution of pore microstructure during coal char activation with steam/CO_2_ mixtures. Carbon.

[39] Kan Y., Yue Q., Kong J., Gao B., Li Q. (2015). The application of activated carbon produced from waste printed circuit boards (PCBs) by H_3_PO_4_ and steam activation for the removal of malachite green. Chem. Eng. J..

[40] Guedidi H., Reinert L., Lévêque J.-M., Soneda Y., Bellakhal N., Duclaux L. (2013). The effects of the surface oxidation of activated carbon, the solution pH and the temperature on adsorption of ibuprofen. Carbon.

[41] Jaria G., Lourenço M.A.O., Silva C.P., Ferreira P., Otero M., Calisto V., Esteves V.I. (2019). Effect of the surface functionalization of a waste-derived activated carbon on pharmaceuticals’ adsorption from water. J. Mol. Liq..

[42] Pakuła M., Walczyk M., Biniak S., Świątkowski A. (2007). Electrochemical and FTIR studies of the mutual influence of lead(II) or iron(III) and phenol on their adsorption from aqueous acid solution by modified activated carbons. Chemosphere.

[43] Sulaymon A.H., Abbood D.W., Ali A.H. (2013). A comparative adsorption/biosorption for the removal of phenol and lead onto granular activated carbon and dried anaerobic sludge. Desalination Water Treat..

[44] Gökçe Y., Aktaş Z. (2014). Nitric acid modification of activated carbon produced from waste tea and adsorption of methylene blue and phenol. Appl. Surf. Sci..

[45] Tran H.N., Huang F.-C., Lee C.-K., Chao H.-P. (2017). Activated carbon derived from spherical hydrochar functionalized with triethylenetetramine: Synthesis, characterizations, and adsorption application. Green Process. Synth..

[46] Alade A.O., Amuda O.S., Ogunleye O.O., Okoya A.A. (2012). Evaluation of interaction of carbonization temperatures and concentrations on the adsorption capacities and removal efficiencies of activated carbons using response surface methodology (RSM). J. Bioremed. Biodegrad..

[47] Montgomery D.C. (2013). Design and Analysis of Experiments.

[48] Habeeb O.A., Kanthasamy R., Ali G.A.M., Yunus R.M. (2017). Application of response surface methodology for optimization of palm kernel shell activated carbon preparation factors for removal of H_2_S from industrial wastewater. J. Teknol..

[49] (2013). SOLID MINERAL FUEL Determination of Ash (ISO 1171:2010 Solid Mineral Fuels—Determination of Ash (MOD)). Prepared by the Federal State Unitary Enterprise “All-Russian Scientific Research Center for Standardization, Information and Certification of Raw Materials, Materials and Substances” (FSUE “VNITSMV”) on the Basis of Its Own Authentic Translation into Russian of the Standard Specified in Paragraph 4.

[50] (2013). Coal—Determination of Bulk Density (ISO 23499:2008, MOD). Developed by the Federal State Unitary Enterprise All-Russian Scientific Research Center for Standardization, Information and Certification of Raw Materials, Materials and Substances (FSUE VNITSMV) on the Basis of Its Own Authentic Translation into Russian of the International Standard Specified in Paragraph 5.

